# Rheological Properties of Fish and Mammalian Gelatin Hydrogels as Bases for Potential Practical Formulations

**DOI:** 10.3390/gels10080486

**Published:** 2024-07-23

**Authors:** Svetlana R. Derkach, Nikolay G. Voron’ko, Yulia A. Kuchina, Daria S. Kolotova, Vladimir A. Grokhovsky, Alena A. Nikiforova, Igor A. Sedov, Dzhigangir A. Faizullin, Yuriy F. Zuev

**Affiliations:** 1 Laboratory of Chemistry and Technology of Marine Bioresources, Institute of Natural Science and Technology, Murmansk Arctic University, Sportivnaya Str. 13, 183010 Murmansk, Russia; voronkonikolay@mail.ru (N.G.V.); kuchinayua@mstu.edu.ru (Y.A.K.); kolotovads@mstu.edu.ru (D.S.K.); grokhovskiyva@mstu.edu.ru (V.A.G.); 2 Kazan Institute of Biochemistry and Biophysics, FRC Kazan Scientific Center of RAS, Lobachevsky Str. 2/31, 420111 Kazan, Russia; alnikiforova22@gmail.com (A.A.N.); igor_sedov@inbox.ru (I.A.S.); dfaizullin@mail.ru (D.A.F.); 3 Institute of Chemistry, Kazan Federal University, Kremlyovskaya Str. 18, 420008 Kazan, Russia

**Keywords:** gelatin, fish, mammalians, hydrogel, rheological properties, molecular weight distribution, amino acids, melting temperature, structure

## Abstract

Hydrogels have the ability to retain large amounts of water within their three-dimensional polymer matrices. These attractive materials are used in medicine and the food industry; they can serve as the basis for structured food products, additives, and various ingredients. Gelatin is one of widely used biopolymers to create hydrogels that exhibit biocompatibility and tunable rheological properties. In this study, we offer a comparative analysis of rheological properties of gelatin-based hydrogels (C = 6.67%), including mammalian gelatins from bovine and porcine skins and fish gelatins from commercial samples and samples extracted from Atlantic cod skin. Mammalian gelatins provide high strength and elasticity to hydrogels. Their melting point lies in the range from 22 to 34 °C. Fish gelatin from cod skin also provides a high strength to hydrogels. Commercial fish gelatin forms weak gels exhibiting low viscoelastic properties and strength, as well as low thermal stability with a melting point of 7 °C. Gelatins were characterized basing on the analysis of amino acid composition, molecular weight distribution, and biopolymer secondary structure in gels. Our research provides a unique rheological comparison of mammalian and fish gelatin hydrogels as a tool for the re-evaluation of fish skin gelatin produced through circular processes.

## 1. Introduction

Gelatin is obtained from fibrillar protein collagen, which forms the basis of body connective tissue and bones. Gelatin is one of most widespread gelling agents in the food industry [1,2,3,4], due to its natural origin, biocompatibility, and relatively low cost. In addition to the food industry, gelatin gels are widely used in biomedical [5] and tissue engineering [6], cosmetic, and pharmaceutical applications [7,8].

Collagen with a molecular weight of 300 kDa is a right-handed helical rod consisting of three alpha helices with a molecular weight of 100 kDa. The collagen triple helix is stabilized by hydrogen bonds as well as covalent cross-links between alpha chains [9,10,11]. The thermal denaturation of collagen leads to the destruction of hydrogen and covalent bonds and the destabilization of the triple helix. As a result, one can obtain gelatin, which is a mixture of polypeptides with different molecular weights; the molecular weight distribution depends significantly on the source and production process [12,13].

When collagen is heat-treated in an acidic or alkaline environment, type A or type B gelatin are obtained, respectively. Type A gelatins have a higher isoelectric point compared to type B, typically 7.5–8.5 versus 4.8–5.2, respectively [7]. The amino acid composition of gelatin is very close to that of collagen, the polypeptide chains of which consist of amino acid triads Gly-X-Y and approximately 50–60% of them contain Pro (X) and Hyp (Y) [14,15], which are responsible for stabilizing the triple helix through the hydrogen bond formation [16]. Along with the amino acid composition, the molecular weight and molecular weight distribution of biopolymers affect the rheological properties of gels. The higher the molecular weight, the stronger the gelatin gel [17].

An important factor determining gelatin gelation is the temperature, since polypeptide chains of gelatin at a temperature below the coil–helix transition are capable of partial renaturation of collagen-like triple helices [11,18,19], acting as junction zones in the spatial network of gel [20,21,22]. The effect of thermal history on the melting/gelation of gelatin gels also strongly affects the gel features [23]. The issues of gelatin gelation and the properties of the resulting gels, which are extremely important for various technological applications, are considered in a large number of fundamental and applied studies, summarized, in particular, in reviews [24,25,26]. To study gelatins, a wide range of physicochemical methods are used, in which rheological research occupies an important place [27,28].

The prevalent sources of gelatin are mammals, namely pork and bovine skin and bones [7]. In recent years, the industry has shown great interest in fish raw materials as a source of collagen [29,30,31]. One of the reasons is related to religious restrictions in the use of animal food products (halal and kosher) [15]. Another, no less important, reason is due to the fact that the fishing industry produces a large amount of waste (skin, scales, bones, and offal) [32] when producing filleted fish products. It must be emphasized that waste recycling is an important problem not only from an industrial but also from an environmental point of view. A tradition of cod fisheries and codfish processing is successfully developing in the northwestern regions of Russia [33]. Our strategic location provides unique access to fish by-products and fish gelatin production, as well as an excellent opportunity to explore them for technological use, in particular for food purposes and novel practical formulations.

The purpose of this work was to study the rheological properties of the gelatin hydrogels that we extracted from Atlantic cod skin in comparison to commercial mammalian gelatins from bovine and porcine skin and commercial fish gelatin from cold-water fish. Particular attention was paid to the kinetics of gelation and the thermal stability of hydrogels. To explain the obtained structural and rheological characteristics, we additionally studied the physicochemical properties of gelatin, namely the amino acid composition, molecular weight distribution, and protein secondary structure. The presented work is a continuation of our previous research [34], which analyzed similarities and differences in the properties of gelatins from different sources.

## 2. Results and Discussion

### 2.1. Rheological Properties of Fish and Mammalian Gelatin Hydrogels

#### 2.1.1. Sol–Gel Transition in the Gelatin–Water System at Constant Temperature

At temperatures above 30 °C, the gelatin polypeptide chain in aqueous solutions is in a random coil conformation. The formation of a low-concentration (C_G_ < 10%) gel at a gelatin concentration exceeding the critical concentration of gelation occurs over time [20,35,36]. When the solution is cooled below 30 °C, the nucleation (renaturation) of sections of collagen-like triple helices occurs as a result of a limited coil–helix conformational transition [18]. The solubility of gelatin decreases and, as a result, aggregates are formed, cross-linking by rigid triple helices. Then, a spatial network emerges from the aggregates; the nodes of this network are sections of the triple helix stabilized by hydrogen bonds [22]. The ability to renature hydrogen-bonded triple-helix junction zones is due to the high content of the amino acid triplet Gly–Pro–Hyp in the polypeptide chain of gelatin (see Section 2.2).

**Figure 1:**
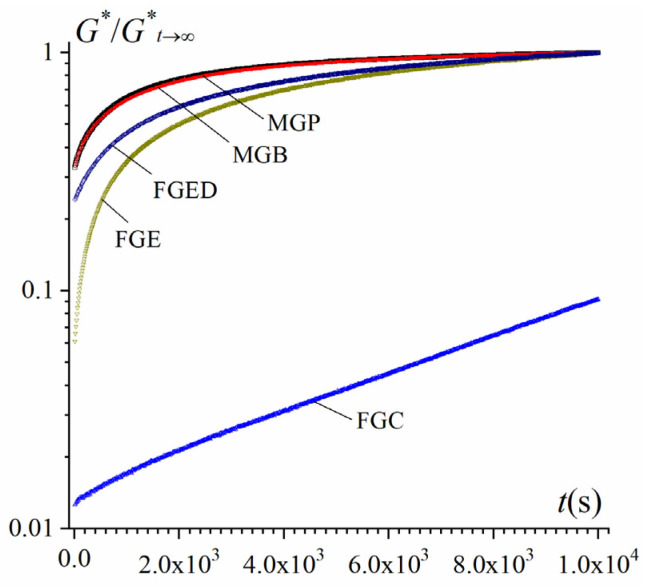
Kinetics of gelation at 4 °C followed by an increase in the dynamic modulus G* normalized by its limiting value, G*_t→∞_. Gelatin tissues are shown in the figure.

Different mammalian and fish gelatin samples exhibit different gelation kinetics. Figure 1 demonstrates that the mammalian gelatins MGP and MGB exhibit high gelation rates under isothermal conditions followed by changes in the time dependence of the complex elastic modulus G*(t). A slightly lower rate of gelation is shown by the fish gelatins FGE and FGED. It should be noted that the commercial fish gelatin FGC does not have time to form a gelatinous spatial network during the experiment (10,000 s).

The kinetics of gelation, considered as a first-order process, are analyzed with the following equation:

(1) $$G^{*}\left(t\right)=G_{\infty }^{*}+\left(G_{0}^{*}-G_{\infty }^{*}\right)Ae^{-kt}$$

where *k* is the gelation rate constant (first-order process) and *A* is the adjustment coefficient. The calculated gelation rate constants for the mammalian gelatins, MGP and MDB, are 6.3·10^−4^ and 5.9·10^−4^ s^−1^, respectively; for the fish gelatins FGE and FGED, the values are two times lower—3.6·10^−4^ and 3.4·10^−4^ s^−1^, respectively. The commercial FGC exhibits a gelation rate constant that is two orders of magnitude lower, 3.4·10^−6^ s^−1^.

#### 2.1.2. Viscoelastic Properties of Gelatin Hydrogels

**Figure 2:**
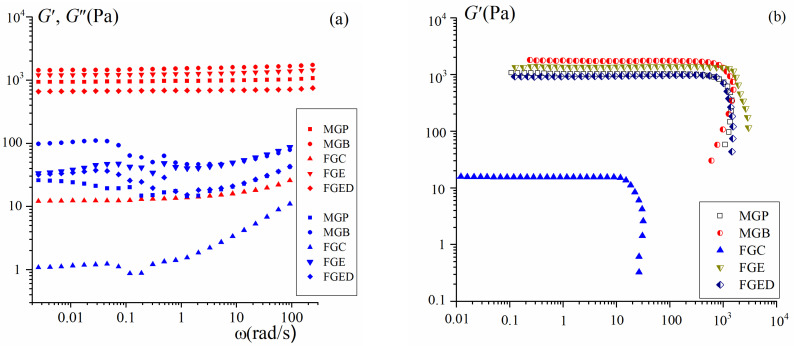
Dependencies of the storage modulus G′ (full points) and the loss modulus G″ (open points) (**a**) on the frequency ω at strain γ = 1% and (**b**) on strain γ at frequency f = 1 Hz for gelatin gels. Gelatin tissues are shown in the figure.

The viscoelastic properties of hydrogels (C_G_ = 6.67%), studied in the dynamic mechanical spectrum mode, are characteristics of physical gels [11,37,38]. Figure 2 shows the frequency independence of the storage modulus over a wide frequency range in the linear region of viscoelastic behavior. In this case, the storage modulus exceeds the loss modulus, G′(ω) > G″(ω), for all gelatin tissues, which indicates the solid-like state of materials [39]. The elastic modulus (rigidity) of gels is comparable in its values to those of the mammalian gelatins MGP and MGB and fish gelatins FGE and FGED and lies in the range from 700 to 1200 Pa. The exception is the weak gel of the commercial FGC, whose elastic modulus is two orders of magnitude lower.

Figure 2b shows stress amplitude scans and the boundary of the linear region in the nonlinear domain of viscoelasticity, characterized by the yield stress, σ_Y_, which makes it possible to classify gelatin hydrogels as soft materials with a yield stress [38,40]. The yield stresses for both mammalian (MGP and MGB) and fish (FGE and FGED) gelatin gels lie in the range of 570–310 Pa. For a weak FGC gel, σ_Y_ is only 9 Pa.

**Figure 3:**
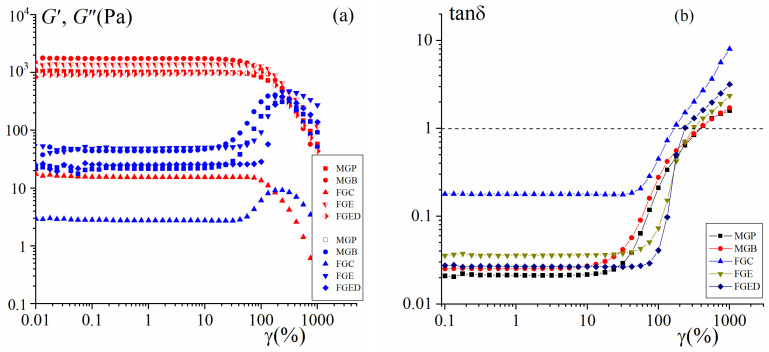
Dependencies of the storage modulus G′ (red points) and the loss modulus G″ (blue points) (**a**,**b**) loss factor tanδ on the strain γ at frequency f = 1 Hz. Gelatin tissues are in the figure.

The results of the viscoelastic properties of gels beyond the linear regime obtained by the method of large amplitude oscillatory shear (LAOS) [41,42] are shown in Figure 3. The deformation amplitude scans (Figure 3a) also show that the elastic behavior of gels dominates over the viscous behavior in the linear–viscoelastic range. The strain sweeps of gelatin gels show a nonlinear threshold between 90% and 1000% strain.

The emergence of a nonlinear response in the viscoelastic behavior with the increase in the strain is demonstrated by a decrease in the storage modulus and an excess of the loss modulus, which passes through a certain maximum (Figure 3a). The observed overshoot of the loss modulus is associated with the partial destruction of the physical bonds in the gel network at high deformations [43]. At a high strain, both G′ and G″ begin to decrease, suggesting that the gelatin gel networks are starting to break and the materials show more a liquid behavior than a solid one.

At some critical deformation, γ*, the values of the two moduli become equal, G′(γ) = G″(γ), and the loss factor, tanδ, is equal to 1 (Figure 3b), which indicates the transition of the samples from the solid to liquid state with the increase in the strain [27,43]. The crossover strain, γ*, for 3% *w*/*w* gelatin gels was detected at a strain of 250%. The highest crossover strain is typical for MGP and MGB, γ* = 420%; for FGE and FGED, γ* was detected at a strain of 310 and 240%, respectively. The lowest value of crossover strain (180%) is shown for FGC, which is associated with a decrease in the stability under vibrational deformation in a weak gel. A similar reaction to deformation scans was observed in gelatin hydrogels obtained from porcine skin [44], bovine skin [45], and codfish [46].

#### 2.1.3. Flow Curves and Yield Stress of Gelatin Gels

**Figure 4:**
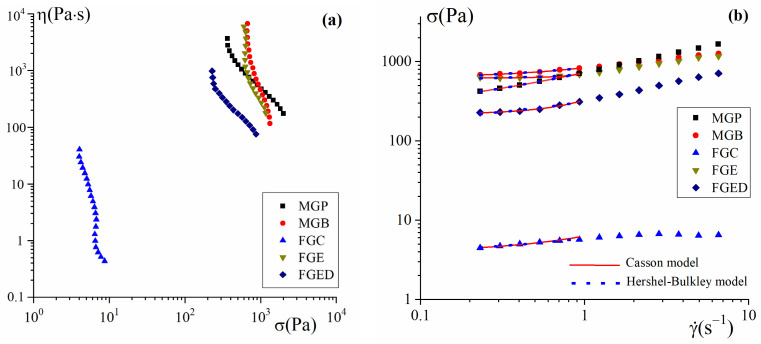
Flow curves η(σ) (**a**) and σ($\dot{\gamma }$) (**b**) of mammalian and fish gelatins.

The nonlinear response in the region of shear stresses exceeding the yield stress, σ > σ_Y_, leads to the steady-state flow of soft materials. Figure 4 shows the flow curves of fish and mammalian gelatin hydrogels, presented as η(σ) and σ($\dot{\gamma }$) in the region above σ_Y_. The shape of the flow curves η(σ) (Figure 4a) corresponds to behavior of viscoplastic materials, for which the apparent viscosity increases without limit with the decrease in the shear stress until transition to the solid state [38].

The observed non-Newtonian behavior, demonstrating a decrease in viscosity with the increase in the shear stress (shear rate) (Figure 4), is due to structural rearrangements of the gelatin physical gel network and the partial destruction of junction zones [27] between polypeptide chains during shear flow. Additionally, the yield stress is usually considered as a characteristic of the gel network strength.

The effect of the molecular weight on the steady flow rheology of salmon skin gelatin showed a significant difference in viscosity [28]. Both the low- and high-molecular-weight gelatins demonstrated a shear-thinning behavior with different apparent viscosity values, which could be explained by the high molecular weight of sample, increasing the probability of gelatin chains joining each other, hence promoting structural stabilization at lower values of shear rate. Similar findings for gelatin from carp scales [47] was associated with changes in the gels’ dispersity and the strength of intermolecular bonds upon various shear rates.

The flow curves of MGP, MGB, and FGE hydrogels lie in the same region σ (and, accordingly, η). The flow curves of FGED lie in the region of slightly lower values of σ and η. In contrast, the FGC hydrogel is significantly different, showing a viscosity that is two orders of magnitude lower. A similar pattern is observed for the yield stress of gels (Figure 4b).

The yield stress was determined by the method of flow curve approximation (Figure 4b) within the Casson (Equation (2)) and Herschel–Bulkley (Equation (3)) rheological models used to describe viscoplastic disperse systems [39,48,49]:

(2) $$\sqrt{\sigma }=\sqrt{\sigma_{Y,C}}+\sqrt{\eta_{p}\dot{\gamma }}$$

(3) $$\sigma =\sigma_{Y,HB}+K{\dot{\gamma }}^{n}$$

where *σ_Y,C_* is the Casson yield stress, *σ_Y,HB_* is the Herschel–Bulkley yield stress, and *η_p_* is the plastic viscosity. The evaluations show that the yield stress decreases in the sequence of MGB > FGE > MGP > FGED > CFG (Figure 4b).

#### 2.1.4. Gel–Sol Transition with the Increase in the Temperature: Thermal Stability of Gels

**Figure 5:**
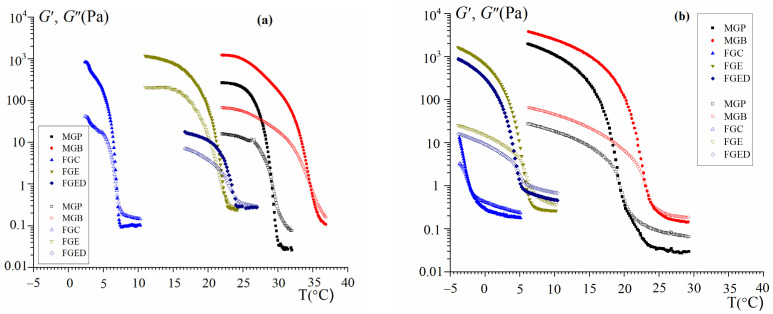
Dependence of storage modulus G′ (full points) and loss modulus G″ (open points) (**a**,**b**) on temperature T for gelatin hydrogels in the heating (**a**) and cooling (**b**) modes. Heating rate (cooling) 1 °C/min; f = 1 Hz; γ = 1%.

The gel–sol transition in systems containing gelatin is thermoreversible and controlled by temperature changes [18,22]. The melting temperature, Tm, and gelation temperature, Tg, are taken to be the crossover point G′(T) = G″(T) on the temperature sweep (Figure 5a,b) with the increase and decrease in the temperature, respectively [27]. The crossover point corresponds to a loss factor value equal to one.

A sharp decrease in G′ with the increase in the temperature (Figure 5a) is associated with the helix–coil conformational transition of gelatin polypeptide chains and the destruction of hydrogen-bonded triple-helix junction zones, accompanied by the complete destruction of the gel network, which turns into a sol. So, Tm can be considered as a characteristic of the gelatin gel thermal stability [11,22,43]. This characteristic is very important when creating various products and materials based on gelatin gels, which must be thermally stable within the room temperature range.

The hysteresis loop characterizing the thermoreversibility of gelatin gels is presented quite clearly in Figure 5a,b. The analysis of the data obtained shows that the MGP and MGB gels are most thermally stable; the Tm values are 34.7 and 29.1 °C, respectively. Somewhat inferior to them are the fish gelatins FGE and FGED, showing Tm values of 23.5 and 22.1 °C, respectively. Similar results were obtained for codfish gelatin in other works reported in the literature [50,51,52]. Commercial fish gelatin exhibits the lowest melting point (6.9 °C).

**Figure 6:**
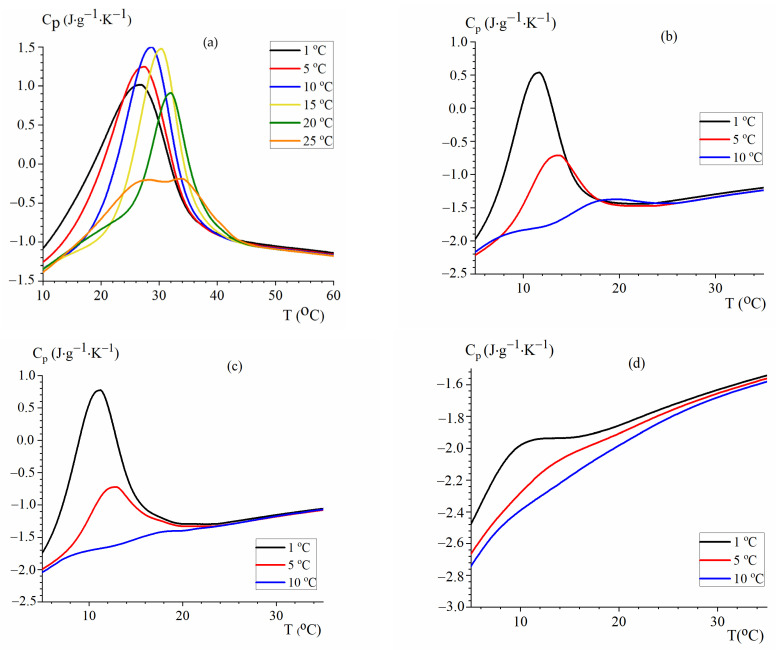
DSC heating thermograms of gelatin gels (C_G_ = 0.5%) formed at different temperatures Tf shown in the legend: (**a**) MGP, (**b**) FGE, (**c**) FGED, and (**d**) FGC.

To study the dependence of the gel–sol transition of mammalian and fish gelatins on the temperature of gel formation, the method of capillary differential scanning calorimetry (DSC) was used. Figure 6 shows the DSC thermograms of gelatin heating after incubation for 5 h at different constant temperatures (Tf) for gel formation. The preliminary experiments showed that this time is enough to achieve the maximum possible gelation, since the enthalpy of melting does not change with further increases in the time of incubation.

A significant increase in the melting (peak maximum) temperature of all gelatins with the increase in the temperature of gel formation (Figure 6a) is observed. The gel–sol transition enthalpy in the case of MGP has a maximum at temperature Tf near 10 °C. At higher temperatures, the yield of the gel likely becomes lower despite its higher thermal stability. A double-humped curve is observed in the thermogram after the incubation of gelatin at 25 °C, which appears to be a sum of two peaks. The peak observed at higher temperatures corresponds to the part of gelatin that gelated at 25 °C, while the low-temperature peak corresponds to the gel formed from the remaining gelatin during its subsequent cooling to 1 °C in order to record the thermogram.

Fish gelatins (Figure 6b–d) have a much lower gel melting point than porcine gelatin. The enthalpy of the gel–sol transition greatly decreases with the increase in Tf, and at Tf = 10 °C, the melting peak becomes almost suppressed. For the two fish gelatin samples FGE and FGED, no significant differences in the parameters of the gel–sol transition are observed (Table 1). The commercial fish gelatin FGC (Figure 6d) has a slightly lower temperature for the gel–sol transition than FGE and FGED, but a much lower enthalpy of transition.

According to [35], at low temperatures of gel formation, the rapid renaturation of triple helices and the association of gelatin chains with the formation of disordered hydrogen bonds occurs. As a consequence, a weak gel network with a low melting point is formed. If gelation occurs at higher temperatures, the renaturation of the helices occurs more slowly, but rigid (rough) networks with a high thermal stability are formed, approaching collagen. Our results fully support these conclusions.

### 2.2. Amino Acid Analysis

The amino acid composition is an important characteristic of gelatin. Gelatin is derived from the destruction and partial hydrolysis of fibrillar protein collagen. Therefore, the amino acid sequence that forms the primary structure can have slight differences according to the type of initial collagen-containing raw material species [13,29] and extraction conditions [53,54].

The ability of gelatin to renature (under temperature decrease) the triple-helical conformation of collagen is explained by the unique sequence of the amino acid triplets Gly-X-Y (where X is usually Pro and Y is often Hyp), which makes up 50–60% of the α-chain [15]. It is this property that explains the unique ability of gelatin of thermoreversible gelation [55]. The collagen-like triple-helix is stabilized by van der Waals interactions between the pyrrolidine rings of Pro and Hyp as well as hydrogen bonds between N-H (Gly), O-H (Hyp), and C=O groups [7]. The content of these amino acids has to be taken into account when discussing the strength of hydrogels.

Table 2 presents the amino acid composition of the mammalian and fish gelatin tissues MGP, MGB, FGC, and FGE. The protein content in the samples ranged from 87.4 to 90.7% (see Section 4.1). It is expected that FGC and FGE exhibit similar amino acid profiles since both samples were derived from the skin of cold-water fish species. For the commercial sample FGC, the Pro-Hyp-Gly contents are 22.48% and 18.62%, and for FGE, they are 19.71% and 18.48%, respectively (Table 2). These results are consistent with the literature data for gelatin from Atlantic salmon [56], dog shark and skipjack tuna [57], cobia [58], tilapia [59], shark (spiny dogfish) [60], and silver carp [61].

It should be noted that the content of imino acids (Pro with Hyp) in mammalian gelatin is higher than in fish gelatins; so, the content of Pro with Hyp is 38.46% in the MGP sample (Table 2). Numerous published data confirm this pattern [29,36,62].

### 2.3. Molecular Weight Distribution

The molecular weight (Mw) as well as the molecular weight distribution (MWD) of biopolymers are important factors influencing rheological (physical and mechanical) properties. The increase in the content of high-molecular-weight fractions provides a higher strength to gelatin gel and increases the thermal stability [63]. The onset of the plastic flow of such gels shifts to higher temperatures. On the contrary, gelatins with a high content of low-molecular-weight fractions show low network strength properties and are characterized generally by worse mechanical and gelling properties [64].

**Figure 7:**
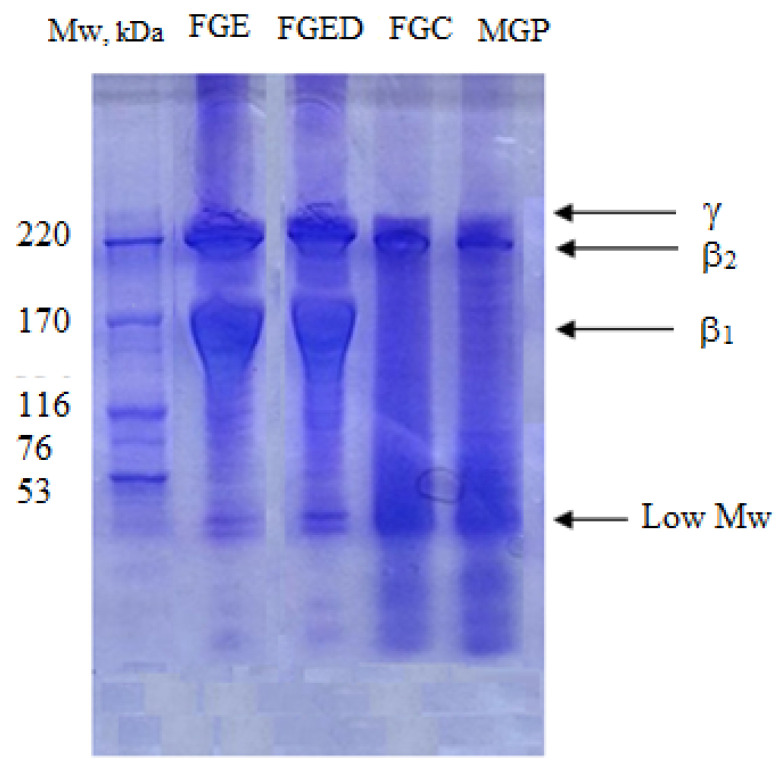
Electropherograms obtained by sodium dodecyl sulphate polyacrylamide gel electrophoresis (SDS-PAGE) for different fish and mammalian gelatins and standard high-molecular-weight markers (GE Healthcare).

The mammalian and fish gelatin samples were analyzed by sodium dodecyl sulphate polyacrylamide gel electrophoresis (SDS-PAGE); the obtained results are shown in Figure 7. On the mammalian (MGP) gelatin electropherogram, it is possible to detect the presence of one intense β-chain at 220 kDa and two weak β-chains at 192 and 172 kDa; there is also a weak monomeric α-chain at 76 kDa (partially hydrolyzed α-chain) and an intense band of low-molecular-weight fractions at Mw < 50 kDa.

A slightly different pattern was observed for fish gelatins. The commercial sample FGC is characterized by the presence of one intense β-chain at 220 kDa and the low-molecular-weight fractions at Mw < 50 kDa. A similar pattern was observed for the fish gelatins FGE and FGED extracted from cod skin. However, it should be noted that these samples show the presence of two different intense dimeric β-chains (β_1_ and β_2_) at 220 and 170 kDa, respectively, and a weak trimeric γ-chain at ~300 kDa. Similar results are known for other fish gelatins [65,66,67,68] obtained, for example, from golden carp [69], trigger fish skin [70], tuna and haddock skins [71], and Atlantic mackerel [72]. A comparative analysis of the gelatins FGE and FGED extracted from the cod by-product (skin) shows that the additional technological operation–purification of gelatin by dialysis against low-molecular inorganic salts does not affect the molecular weight distribution; low-molecular-weight fractions are present in both samples.

For the more accurate determination of the gelatins’ molecular weight as well as molecular weight distribution, we used gel permeation chromatography (GPC). Table 3 shows the molecular weight distribution of the mammalian and codfish skin gelatins, according to the peaks of eluograms presented in the Supplementary Materials (Figure S1), where Rt is the retention time, Mw is the weight-average molecular weight, Mn is the number-average molecular weight, and PDI is the polydispersity index, characterizing the polydispersity of the gelatin samples, PDI = Mw/Mn.

MGP includes a fraction with the average molecular weight Mn ~ 136 kDa and Mw ~ 196 kDa (97.69%), which are compatible with the molecular weight of α- and β-chains, respectively. The viscosity-average molecular weight, Mη, determined by the viscometric method by measuring the viscosity of dilute solutions, is close to these values, Mη = 122 kDa.

FGC contains one fraction with the average molecular weight Mn = 160 kDa and Mw = 227 kDa (100%), while the average viscosity mass Mη = 133 kDa. The two other samples of fish gelatin FGE and FGED also consist mainly of one fraction, with the average molecular weight Mn = 201 ÷ 202 kDa and Mw = 325 ÷ 307 kDa (98–99%), which correspond to the molecular weights of β- and γ-chains, respectively. The viscosity-average molecular weight, Mη, is 145 and 147 kDa, respectively.

The extraction of fish gelatin under mild conditions (pH 5.0) is apparently accompanied by the incomplete destruction of covalent crosslinks between alpha chains (100 kDa) in collagen molecules [61]. As a consequence, the molecular weight distribution of FGE and FGED is characterized by the mixture of dimeric β-chains and trimeric γ-chains. A similar result was obtained in [52] when studying codfish gelatin. The use of asymmetrical flow field-flow fractionation coupled with multi-angle light scattering made it possible to show that, in native gelatin, the α-, β-, and γ-chains can bind to form compounds with a higher molar mass [73].

These data (Table 3) are in agreement with the SDS-PAGE results (Figure 7). In addition, all mammalian and fish gelatins, except FGC, present the lowest peak (1–2%) corresponding to fragments of lower molecular weight (Mw = 19 ÷ 21 kDa, i.e., Mw < 50 kDa).

Obviously, the terminal groups of macromolecules can undergo hydrolysis during extraction in an acidic environment [72,74] or in the presence of enzymes [46]. This leads to appearance of a small amount of low-molecular-weight fractions in the composition of gelatin.

Table 3 shows the polydispersity index (PDI) calculated for each individual peak. For the low-molecular-weight fractions of all gelatins, the PDI is close to the unity, which may indicate a high degree of selective hydrolysis when using various acids [75]. On the other hand, for high-molecular-weight peaks, the PDI is 1.4–1.5; polydispersity may indicate intramolecular cleavage has occurred in the macromolecular chain [73].

### 2.4. Secondary Structure Analysis

**Figure 8:**
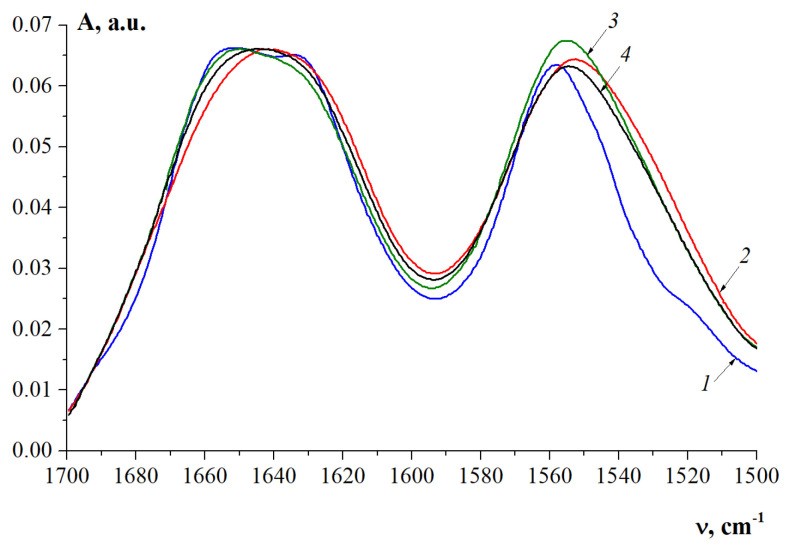
FTIR spectra in the absorption region of Amide I (C=O) and Amide II (N-H) for mammalian MGP (1) and fish FGC (2), FGE (3), and FGED (4) gelatins.

FTIR spectroscopy has been employed to discern the structural and conformational characteristics of fish and mammalian gelatins. The Amide I band is the most sensitive to changes in the protein secondary structure [76,77,78,79]. It is well reported that Amide I is coupled with the C=O stretching vibration connected to the CN stretch and NH bending modes occurring in the range of 1600–1700 cm^−1^ [80,81]. The differences in the spectral pattern within the Amide I region primarily describe the conformational flexibility of gelatin polypeptide chains [82]. Figure 8 shows the FTIR spectra of the studied fish and mammalian gelatins in the Amide I absorption region. The second derivative bands of Amide I are presented in the Supplementary Materials (Figure S2).

To characterize the gelatin secondary structure, we used the Amide I decomposition components, attributable, according to [83,84,85], to various C=O groups of the Gly-Pro-Hyp tripeptide sequence included in the gelatin chain: 1618 cm^−1^, hydrated C=O (Pro); 1630 cm^−1^, hydrated C=O (Pro, Hyp); 1642 cm^−1^, hydrated C=O (Gly); 1660 cm^−1^, C=O (Pro) groups connected by hydrogen bonds with C-N-H (Gly) as part of a triple helix; 1683 and 1692 cm^−1^, beta turns of various types.

In the FTIR spectrum of pork gelatin (Figure 8, curve 1), the components at 1660 cm^−1^ and 1692 cm^−1^ are enhanced, and the components at 1642 and 1683 cm^−1^ are weakened. The amide II band in the spectrum is sharply narrowed due to the weakening of the 1545 cm^–1^ component (N-H group with weak hydrogen bonds).

The analysis of the fine structure of the Amide I band by the second derivative (Figure S2) shows that the MGP spectrum differs from those of the fish gelatins FGC and FGED by a sharp weakening of the components at 1618, 1642, and 1683 cm^−1^, free, hydrated C=O (Pro) and C=O (Gly) groups, indicating a high proportion of triple helices in pork gelatin. A similar structure with a high content of triple helices is present in FGE.

The content of triple helices in the secondary structure of gelatin is an important characteristic, since the level of renaturation of collagen-like structures determines the ability of gelatin to form high-strength gels [4,82]. Thus, a study of gelatin extracted by prior ultrasonication reveals [69] that gelatin has higher gelling and melting temperatures than those produced by conventional methods. FTIR spectra show that such gelatin has higher cross-links, stabilized by hydrogen bonds, which are responsible for stabilizing gelatin triple helices.

To calculate the content of triple collagen-like helices, we decomposed the Amide I band into the components indicated above and corresponding to the absorption bands at 1618, 1630, 1642, 1660, 1683, and 1692 cm^−1^. The hydrated components C=O (Pro, Hyp) and C=O (Gly) can be both within the triple helix and outside it, but are spectrally indistinguishable. So, as a measure of helicity, we used the C=O (Pro) group content associated by hydrogen bonds to N-H (Gly), absorbing at 1660 cm^−1^.

**Figure 9:**
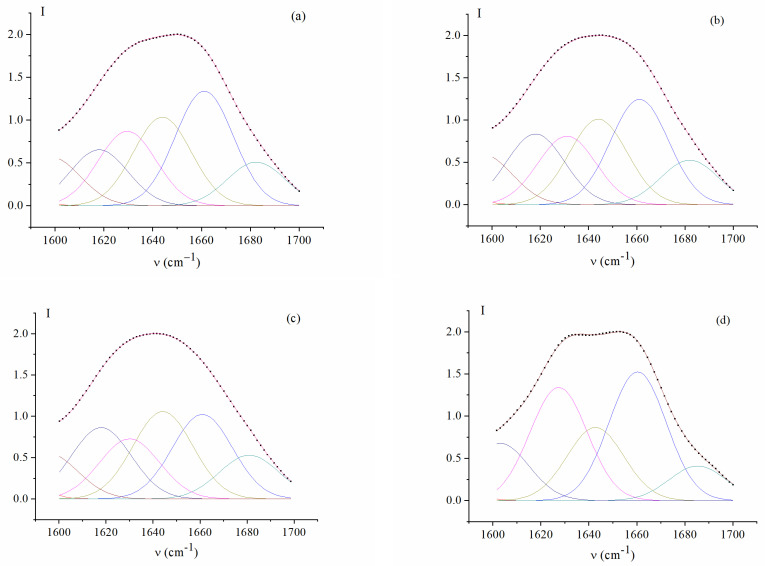
Decomposition of Amide I band into components in the FTIR spectra of gelatin gels: FGE (**a**), FGED (**b**), FGC (**c**), and MGP (**d**).

**Figure 10:**
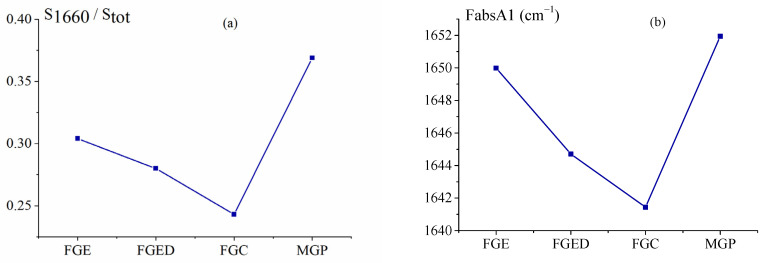
(**a**) Ratio of the I 1660 cm^−1^ component area to the total area of C=O groups in Amide I absorption; (**b**) position of the Amide I maximum absorption band in the gelatin gel FTIR spectra.

It should be noted that the proportion of these groups is proportional to the content of triple helices, but not equal to it. The results of the decompositions are shown in Figure 9. The highest level of renaturation, i.e., the content of triple collagen-like helices, is present in MGP and FGE, obtained from cod skin (Figure 10a).

The increase in the wave number maximum of the Amide I band reflects an increase in the 1660 cm^−1^ component, that is, an increase in the proportion of imide C=O (Pro) hydrogen bonded to N-H (Gly), which is responsible for triple helix stabilization. The Amide I band appears at the wavenumbers of 1649.98 cm^−1^ and 1651.94 cm^−1^ for FGE and MGP, respectively (Figure 10b). These data also confirm the similarity of the secondary structure of fish FGE and pork MGP in terms of the helix content.

### 2.5. Microstructures of Gels

**Figure 11:**
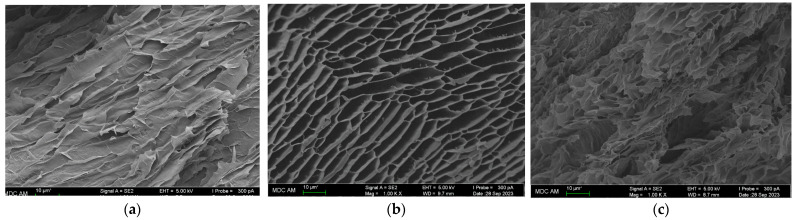
Scanning electronic micrographs of hydrogels from: (**a**) MGP, (**b**) FGC, and (**c**) FGE.

Scanning electron microscopy (SEM) was used for the morphological analysis of gelatin cryo-gel textures. The microstructures of gels from MGP, FGC, and FGE are shown in Figure 11. The porcine gelatin gel (Figure 11a) exhibits the densest structure formed by thick tightly adjacent filaments forming a non-smooth surface with fibrous parts and the absence of porosity. In contrast, the commercial fish gelatin gel (Figure 11b) shows a porous structure formed by thinner filaments with a large volume of pores between them. This result is consistent with those of [34,67], where it is shown that the gel texture depends on the physicochemical properties of gelatin, which are determined by the properties of collagen-containing raw materials as well as the conditions of its processing [66,69,86]. A high porosity is often a characteristic of fish gelatin [72,87,88] extracted using an alkali and/or acid.

The microstructural analysis of the gel from FGE shows a fairly dense structure formed by thick filaments with very small pores (Figure 11c). The texture of this gel is most similar in its characteristics to that of the MGP gel.

### 2.6. Comparative Analysis

The physicochemical properties of various samples of mammalian and fish gelatins were studied and analyzed: gelatin from porcine skin, Type A (MGP); gelatin from bovine skin, Type B (MGB); commercial gelatin from cold-water fish skin (FGC); gelatin extracted from codfish skin (FGE); and the same gelatin tissue purified by dialysis (FGED). We studied the rheological properties of gelatin hydrogels, as well as the kinetics of the sol–gel transition and compared the rate of gelation (Section 2.1.1), viscoelastic properties of the hydrogels, elastic modulus (Section 2.1.2), yield stress (Section 2.1.3), and thermal stability and melting enthalpies (Section 2.1.4).

The comparative analysis showed that the most elastic hydrogels and those with the highest strength are those formed by bovine and porcine gelatins. This result is not surprising; considerable literature data confirm the presence of higher rheological characteristics and melting temperatures in mammalian gelatins compared to fish gelatins [13,62,89].

It is significant that the fish gelatin FGE, extracted by us from cod skin under mild conditions (pH 5.0) [63], in terms of rheological characteristics, is close to mammalian gelatins. The purification of fish gelatin by dialysis leads to a decrease in the hydrogel strength and elasticity and practically does not change its sol–gel transition temperature. FGC forms a weak gel with a low elastic modulus (Table 4). Thus, the strength and elasticity of the gel decreases in the order of MGB > MGP > FGE > FGED > CFG.

Based on the data presented above, our previous studies [45,46,63], and data published in the literature [27,29], the following justification for the differences in the rheological properties of fish and mammalian gelatins is proposed, taking into account the microscopic properties, amino acid composition (Section 2.2), molecular weight distribution (Section 2.3), secondary structure of gelatins (Section 2.4), and microstructure of the gels (Section 2.5).

The model of the physical network of gelatin gels is a combination of structural chain elements: elastic (triple collagen-like helix-junction zones) and viscous (disordered polypeptide sections of the chain), constituting an infinite cluster [35] with its fractal dimension [90] characterized by a set of rheological parameters.

Among all the studied samples, the mammalian gelatins MGP and MGB contain the highest amount of imino acids (proline and hydroxyproline) responsible for the renaturation of triple helices (see Results Section 2.2) and the highest content of triple helices among secondary structure conformations according to IR spectroscopy (see Results Section 2.4). This leads to the formation of a large number of strong junction zones in the structural network of the gel [29], which is revealed by high rheological parameters (strength, elastic modulus, and yield strength) and high thermal stability (melting point and melting enthalpy).

The fish gelatin FGE has a lower proportion of Gly+Pro+Hyp in its amino acid composition (see Results Section 2.2) compared to the mammalian gelatin. However, FGE possesses mainly high-molecular-weight fractions (see Results Section 2.3), which impart higher strength properties and increased hardness and thermal stability to the biopolymer [14]. The content of triple helices as an element of the secondary structure is comparable to the same parameter in porcine gelatin. As a result, the rheological characteristics and thermal stability of the FGE gel are only slightly inferior to those of the MGP and MGB gels.

In contrast, the commercial tissue FGC forms weak gels with a low thermal stability, low gelation rate constant, and low gelation enthalpy, which are generally characteristic of cold-water fish gelatin [29]. This is not surprising [91], due to the lower imino acid contents (see Results Section 2.2), which, in turn, reduces the propensity for intermolecular triple-helix formation (see Results Section 2.4) in gelatin.

The rheological properties discussed are determined by the structural properties of gels. The SEM studies (see Results Section 2.5) confirm that both strong MGP and FGE gels have a finely porous, dense microstructure. In contrast, weak FGC gels are highly porous, and their gel network is formed by thin threads with a large number of voids between them.

## 3. Conclusions

The growing demand for fish gelatins around the world, caused by searches for novel alimentary sources, explains the development of further research and study of fish gelatins as an alternative to mammalian ones. In recent years, significant progress has been made in the development of technologies for extracting gelatin from collagen-containing fish raw materials (skin, bones, and by-products). Increasing the production of fish gelatin also solves the environmental problem of creating waste-free fish processing industries. The unique ability of gelatin of thermoreversible gelation determines its demand in biomedicine, pharmaceuticals, and the food industry, including meeting the demand in the kosher market. Gelatin hydrogels are an excellent basis for novel practical formulations. In this case, the rheological properties of hydrogels must satisfy certain requirements.

In this work, which is a continuation of previous research [34], comprehensive studies of the rheological properties of gelatin hydrogels obtained from various raw materials were carried out. A comparative analysis of gels of fish gelatin, extracted by us from the skin of Atlantic cod under mild conditions, not subjected to and subjected to additional purification by dialysis, with gels of commercial mammalian and fish gelatin was carried out.

Our rheological results have shown that the gelatin extracted from codfish skin forms hydrogels that are similar in their basic characteristics (strength, elastic modulus, melting point, and gelation rate) to mammalian ones and is significantly superior to commercial gelatin from cold-water fish. Moreover, the SEM microstructural analysis revealed the high network density of gelatin from Atlantic codfish skin and mammalian gelatin, as well as the highly porous gel structure of commercial fish gelatin. The purification of gelatin from codfish skin by dialysis did not lead to hydrogel strengthening. The rheological properties of gelatin are determined by its physicochemical and structural characteristics. This work examined in detail the amino acid composition, molecular weight distribution, and secondary structure of gelatins. It was shown that gelatin from Atlantic codfish skin contains the amino acids Gly+Pro+Hyp in smaller quantities than the mammalian gelatin, while being characterized by a high content of high-molecular-weight fractions and number of alpha helices in comparison to the mammalian one.

The results obtained can be useful to and applied by processors to control the physicochemical parameters of fish gelatin at the extraction stage and then obtain hydrogels with the necessary rheological characteristics when creating novel practical formulations. Moreover, improving these characteristics is also possible through physical modifications [62], such as the introduction of polysaccharides, as well as chemical modification [29] and the use of cross-linking agents.

## 4. Materials and Methods

### 4.1. Materials

Three gelatin tissues from Sigma-Aldrich Corp. (Saint Louis, MO, USA) were used:

1. MGP—mammalian gelatin from porcine skin (Type A) G6144, Lot No BCBR5299V, Switzerland;
2. MGB—mammalian gelatin from bovine skin (Type B) G9382, Lot No SLBM7200V, USA;
3. FGC—fish gelatin (commercial) from cold-water fish skin G7041, Lot No SLCC7087, Canada.

In addition, two more samples of fish gelatin were used:

4. FGE—fish gelatin extracted (at pH 5.0) from codfish skin [34];
5. FGED—fish gelatin extracted (at pH 5.0) from codfish skin and then dialyzed.

The technological scheme for obtaining gelatin from codfish skin by the thermal destruction method in acidic media is shown in Figure S3. The isoelectric points (pI) (determined by the viscometric and turbidimetric methods) for various gelatins were equal to: 8.1 (MGP), 4.7 (MGB), 7.6 (FGC), 7.1 (FGE), and 7.1 (FGED). The chemical composition of the gelatins is presented in Table 5.

### 4.2. Preparation of Hydrogels

Hydrogels for rheological experiments were obtained from gelatin solutions (C_G_ = 6.67%) by cooling at 4 °C for 20 h. To prepare an aqueous solution of gelatin, a sample of a given weight swelled in distillated water at 20 °C for 1 h. Then, it was dissolved at the elevated temperature of 50 °C.

### 4.3. Determination of Chemical Composition

The content of moisture, fat, total nitrogen, and minerals was determined according to standard methods. The moisture content in the samples was determined after drying them to a constant weight at t = 105 ± 5 °C. The amount of fat was determined by the Soxhlet method (solvent extraction), total nitrogen by the Kjeldahl method, and mineral substances by the method of burning samples in a muffle sword at T = 550 ± 10 °C.

### 4.4. Rheological Methods

The rheological characteristics of hydrogels were studied under shear deformation using a compact rheometer Physica MCR 302 (Anton Paar GmbH, Graz, Austria) with the cone–plane measuring system CP50-1 (diameter of 50 mm, angle of 1°, and gap between the top of the cone and the plane of 0.100 mm). The experimental protocols were set as follows:

1. Time dependence of the complex modulus (G*) at a constant frequency (f = 1 Hz; ω = 2π rad/s) and deformation amplitude (γ = 1%) and initial temperature of 30.0 °C;
2. Periodic oscillations with a deformation amplitude (γ) in the range of 10^−2^–10^3^% at the constant f = 1 Hz;
3. Periodic oscillations with frequency (ω) in the range of 10^−3^–10^2^ rad/s at γ = 1%;
4. Shear rate ($\dot{\gamma }$) control mode in the range of 10^−1^–10^2^ s^−1^;
5. Temperature scanning in the heating and cooling modes with a ramp rate of 1 °C/min at the constant f = 1 Hz and γ = 1%.

The experimental protocols (1–4) were performed at the temperature of 4.00 ± 0.03 °C. When studying the kinetics of gelation (Section 2.1.1), the procedure was conducted as follows. A gelatin solution was prepared at 50 °C, cooled to 30 °C, and then placed in a thermostated measuring cell of the rheometer, quickly cooled to 4 °C; then, measurements were carried out at the temperature of 4 °C.

The relative error in measuring the rheological parameters did not exceed 10%. The reproducibility of the results of the rheological measurements was controlled by the parallel testing of two samples with the same content.

### 4.5. GPC

The determination of the molecular weight distribution of the gelatins was carried out by gel-permeation chromatography (GPC) using a high-performance liquid chromatograph LC-20AD (Shimadzu, Kyoto, Japan). The analysis conditions were as follows: eluent of 0.5 N acetic acid solution; flow rate of 0.8 mL/min; T = 40 °C; ELSD detector (low-temperature evaporative light-scattering detector); and column: TSK-GEL G3000SWXL, 7.8 mm ID × 30.0 cm L, 5 μm.

### 4.6. SDS-PAGE

Sodium dodecyl sulphate polyacrylamide gel electrophoresis (SDS-PAGE) was conducted to determine the gelatin molecular weight distribution. Electropherograms were obtained on a Mini-PROTEAN Tetra System (BIO-RAD, Berkeley, CA, USA); standard polyacrylamide Smart PAGETM Protein Gel Plus gradient-filled polyacrylamide plates with a gel concentration from 4 to 20% (Smart-Lifesciences) were used for protein separation.

A gelatin solution (1.0%) was mixed with a buffer solution at a volume ratio of 1:1, kept at a temperature of 95 °C for 5 min, and placed on a polyacrylamide plate. The electrophoretic separation of protein fractions was carried out at room temperature, with the following separation conditions: current of 40 mA and voltage of 100 V. After the completion of electrophoresis, the gel plate was placed for 30 min in an acetic–alcohol solution (10% acetic acid and 40% ethyl alcohol) for fixation, and then, the proteins were stained using the Coomassie method at 20–25 °C for 60 min at 10%, with the acetic acid solution containing 1.25 g/L Phast Gel Blue. Then, the plate was washed in 10% acetic acid solution at room temperature.

The molecular weight of the protein fractions was determined using a calibration graph constructed relative to the mobility of the standard markers of proteins with a known molecular weight from 220 kDa (Myosin) to 53 kDa (Glutamic dehydrogenase) from GE Healthcare, (Chalfont St. Giles, UK).

### 4.7. DSC

The melting temperature and enthalpy of gels was determined by capillary differential scanning calorimetry (DSC) using a NanoDSC calorimeter (TA Instruments, New Castle, DE, USA). The calorimeter measuring cell with a volume of 300 μL was filled with a 0.5% gelatin solution at a temperature of 25 °C. The solution was thoroughly degassed with continuous stirring in vacuum to prevent the formation of bubbles inside the cell. The reference cell was filled with distilled and degassed water. During the experiments, the gelatin solution in the calorimeter cell was first heated from 25 to 60 °C at a constant rate of 2 K/min to disrupt the gel, then cooled at a rate of 10 K/min to the desired temperature, and kept at this temperature for 5 h to form a gel. Then, it was cooled down to 1 °C and immediately heated at a rate of 2 K/min (pork gelatin) or 1 K/min (fish gelatin) to 60 °C. During heating, a gel–sol transition of gelatin occurred, which produced a peak in the DSC thermogram. To determine the peak maximum temperature and area, the sigmoidal baseline was subtracted from the heat flux versus the cell temperature curve. The enthalpy of transition was determined by the integration of the resulting peak.

### 4.8. ATR-FTIR Spectroscopy

An attenuated total reflectance Fourier transform infrared (ATR-FTIR) spectroscopic analysis was conducted to study the gelatin secondary structure in gels. The spectra were registered using a FTIR spectrometer Invenio S (Bruker) equipped with the attenuated total reflection (ATR) accessory with a triple-bounced ZnSe crystal. The spectra were collected at a 4 cm^−1^ resolution, accumulating 128 scans in the range of 1700–1500 cm^−1^ in the absorption region of Amide I and Amide II. The gel samples (gelatin concentration of 6.67%) were placed on the surface of the ATR crystal and thermostated at 4 °C. The resulting spectra were corrected for solvent water and atmospheric water vapor absorption. The spectra were processed and the second derivative was calculated using the OPUS 7.0 program (Bruker Optik GmbH 2012, Ettlingen, Germany).

The Amide I spectrum was decomposed into components using the fityk 1.3.1 program (http://fityk.sharewarejunction.com/ (accessed on 3 June 2024). Visually distinguishable components of the Amide I second-derivative spectra were used as an initial approximation for the number and position of the components. The following set of components was used: 1618, 1630, 1642, 1660, 1682, and 1693 cm^−1^, due to the absorption of C=O groups. To adjust the non-peptide absorption at the edges of the Amide I band, additional components at 1567 and 1600 cm^−1^ were used. During the fitting process, the position and half-width of the components could vary within ±(1.0–1.2) cm^–1^.

### 4.9. SEM

Scanning electron microscopy (SEM) was used to analyze the morphology of the gelatin gels using the field emission scanning electron microscope “Merlin” (“Carl Zeiss”, Oberkochen, Germany). The electron microscopy experiments were carried out using gelatin cryogels prepared as follows. Gelatin hydrogels (concentration of 10%) were frozen in liquid nitrogen, snapped immediately, and vacuum freeze-dried to obtain xerogels. The fractured sections were coated with gold for SEM observations. The morphology was studied at the accelerating voltage of 15 kV.

## Figures and Tables

**Table 1:** Peak maximum temperatures, tmax, and melting enthalpies, ∆H, of gelatin gels formed at different temperatures.

Gelatin Tissue	t_max_ (°C)			∆H (J·g^–1^)		
	1 °C	5 °C	10 °C	1 °C	5 °C	10 °C
MGP	27.0	27.5	28.3	22.2	25.1	25.6
FGE	11.4	13.8	18.4	12.3	4.6	1.6
FGED	11.2	12.4	18.3	12.7	3.0	0.5
FGC	10.8	13.5	17.4	1.0	0.2	0.1

**Table 2:** Amino acid content of commercial pork gelatin, MGP, commercial fish gelatin from cold water, FGC, and gelatin recovered from codfish skins, FGE (% or g/100 g protein).

Amino Acids	Amino Acids	MGP	MGB	FGC	FGE
Glycine	Gly	23.24 ± 1.16	22.87 ± 1.14	18.62 ± 0.93	18.48 ± 0.92
Proline	Pro	21.30 ± 1.06	19.96 ± 1.00	12.90 ± 0.64	12.25 ± 0.61
Hydroxyproline	Hyp	17.16 ± 0.86	18.58 ± 0.93	9.58 ± 0.48	7.46 ± 0.37
Aspartic acid	Asp	6.04 ± 0.30	5.17 ± 0.26	5.62 ± 0.28	5.57 ± 0.28
Glutamic acid	Glu	10.81 ± 0.54	10.09 ± 0.50	9.31 ± 0.47	9.13 ± 0.46
Serine	Ser	3.95 ± 0.20	3.93 ± 0.20	6.36 ± 0.32	6.57 ± 0.33
Histidine	His	1.46 ± 0.07	0.95 ± 0.05	1.66 ± 0.08	1.89 ± 0.09
Threonine	Thr	1.61 ± 0.08	1.59 ± 0.08	2.58 ± 0.13	2.67 ± 0.13
Arginine	Arg	7.87 ± 0.39	7.94 ± 0.40	7.65 ± 0.38	7.68 ± 0.38
Alanine	Ala	10.14 ± 0.51	10.62 ± 0.53	9.40 ± 0.47	9.35 ± 0.47
Taurine	Tau	-	0,67 ± 0.03	2.86 ± 0.14	3.67 ± 0.18
Tyrosine	Tyr	0.86 ± 0.04	0.93 ± 0.05	0.83 ± 0.04	1.00 ± 0.05
Valine	Val	2.65 ± 0.13	2.57 ± 0.13	2.12 ± 0.11	2.12 ± 0.11
Methionine	Met	0.80 ± 0.04	0.26 ± 0.01	1.57 ± 0.08	1.78 ± 0.09
Isoleucine	Ile	1.57 ± 0.08	1.75 ± 0.09	1.47 ± 0.07	1.56 ± 0.08
Leucine	Leu	3.43 ± 0.17	3.50 ± 0.17	2.76 ± 0.14	2.90 ± 0.14
Lysine	Lys	2.26 ± 0.11	1.84 ± 0.09	2.30 ± 0.12	3.56 ± 0.18
Phenylalanine	Phe	2.24 ± 0.11	2.25 ± 0.11	2.40 ± 0.12	2.34 ± 0.12

Errors are the confidence intervals for *n* = 2 (replicates of independent batches) and α = 0.05.

**Table 3:** Molecular weight distribution of mammalian and codfish skin gelatins.

Gelatin Tissue	Peak Number	Rt, min	Mn, kDa	Mw, kDa	PDI	Peak Area, %
MGP	1	7.9 ± 0.0	136.28 ± 11.3	196.08 ± 10.6	1.439 ± 0.009	97.69 ± 2.15
	2	11.9 ± 0.0	24.75 ± 0.9	24.78 ± 0.9	1.001 ± 0.000	1.25 ± 0.04
	3	12.4 ± 0.0	21.54 ± 0.8	21.60 ± 0.8	1.003 ± 0.000	1.06 ± 0.03
FGC	1	7.7 ± 0.0	160.11 ± 15.2	227.55 ± 13.9	1.421 ± 0.008	100.0 ± 0.0
FGE	1	7.5 ± 0.0	201.80 ± 19.1	325.17 ± 13.9	1.611 ± 0.007	98.37 ± 1.52
	2	12.5 ± 0.0	18.90 ± 0.9	19.51 ± 1.0	1.032 ± 0.001	1.63 ± 0.06
FGED	1	7.5 ± 0.0	202.26 ± 18.0	306.71 ± 12.3	1.516 ± 0.006	99.26 ± 0.08
	2	12.6 ± 0.0	20.10 ± 0.8	20.28 ± 0.8	1.009 ± 0.000	0.74 ± 0.02

Values are represented as mean ± confidence intervals (for *n* = 2 and α = 0.05).

**Table 4:** Molecular weight distribution of mammalian and codfish skin gelatins.

Gelatin Tissue	G′_pl_, Pa	γ^*^, %	σ_Y_, Pa	σ_Y,C_, Pa	σ_Y,HB_, Pa	T_m_, °C	T_g_, °C
MGB	1430 ± 70	420 ± 20	518 ± 32	544 ± 33	631 ± 34	34.7 ± 0.9	23.3 ± 0.6
MGP	940 ± 50	420 ± 30	317 ± 21	209 ± 22	227 ± 25	29.1 ± 0.8	19.3 ± 0.5
FGE	1270 ± 60	310 ± 20	572 ± 35	554 ± 35	623 ± 30	22.1 ± 0.4	5.5 ± 0.4
FGED	670 ± 40	240 ± 10	309 ± 25	146 ± 15	218 ± 15	23.5 ± 0.5	4.5 ± 0.4
FGC	12.2 ± 1.0	180 ± 10	8.9 ± 0.8	3.5 ± 0.3	4.0 ± 0.4	6.9 ± 0.7	−2.3 ± 0.3

**Table 5:** Rheological characteristics of hydrogels from mammalian and fish gelatins.

Characteristics	MGP	MGB	FGC	FGE	FGED
Moisture content, X, %	12.0 ± 0.2	9.0 ± 0.2	12.6 ± 0.2	8.0 ± 0.2	7.8 ± 0.2
Total nitrogen, N_T_, %	16.0 ± 0.1	16.0 ± 0.1	15.9 ± 0.1	16.5 ± 0.1	16.6 ± 0.1
Protein P, %	88.0 ± 0.5	89.7 ± 0.5	87.4 ± 0.5	90.7 ± 0.5	91.3 ± 0.5
Ash, %	0.10 ± 0.05	1.20 ± 0.10	-	1.50 ± 0.10	1.10 ± 0.10

## Data Availability

Data are contained within the article.

## Supplementary Materials

The following supporting information can be downloaded at: https://www.mdpi.com/article/10.3390/gels10080486/s1. Figure S1: Molecular weight distribution according to the peaks of the eluograms; Figure S2: The second-derivative bands of Amide I; Figure S3: Technological scheme for obtaining codfish gelatin.
