# Supplementary material for: Rheological Properties of Fish and Mammalian Gelatin Hydrogels as Bases for Potential Practical Formulations
Source: Gels. 2024 Jul 23;10(8):486. doi: 10.3390/gels10080486 (PMC11354070; doi:10.3390/gels10080486)
Supplement: Supplementary file 1 [file gels-10-00486-s001.zip › gels-3112612-supplementary.pptx]

## Slide 1
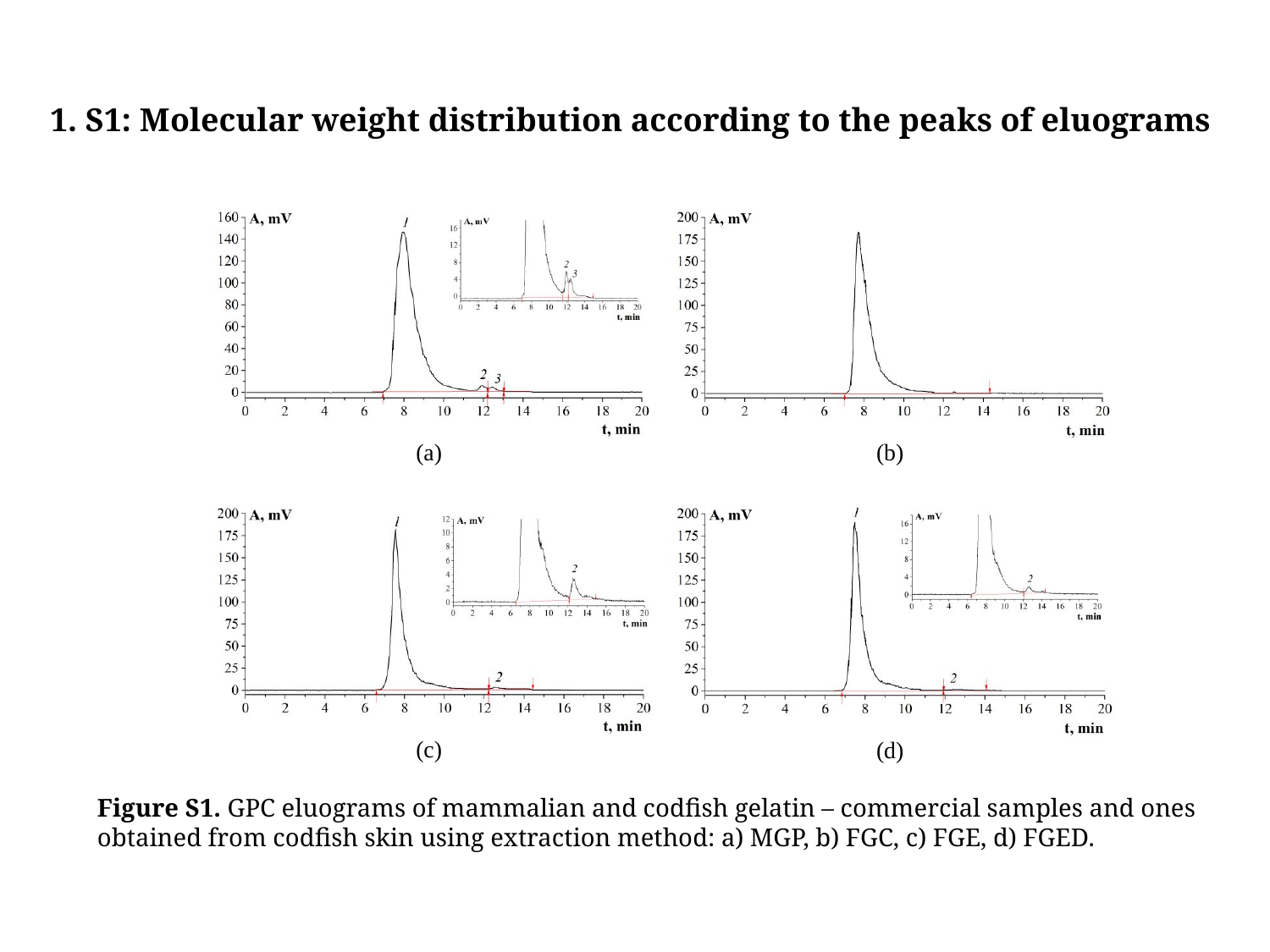

1. S1: Molecular weight distribution according to the peaks of eluograms
Figure S1. GPC eluograms of mammalian and codfish gelatin – commercial samples and ones obtained from codfish skin using extraction method: a) MGP, b) FGC, c) FGE, d) FGED.

## Slide 2
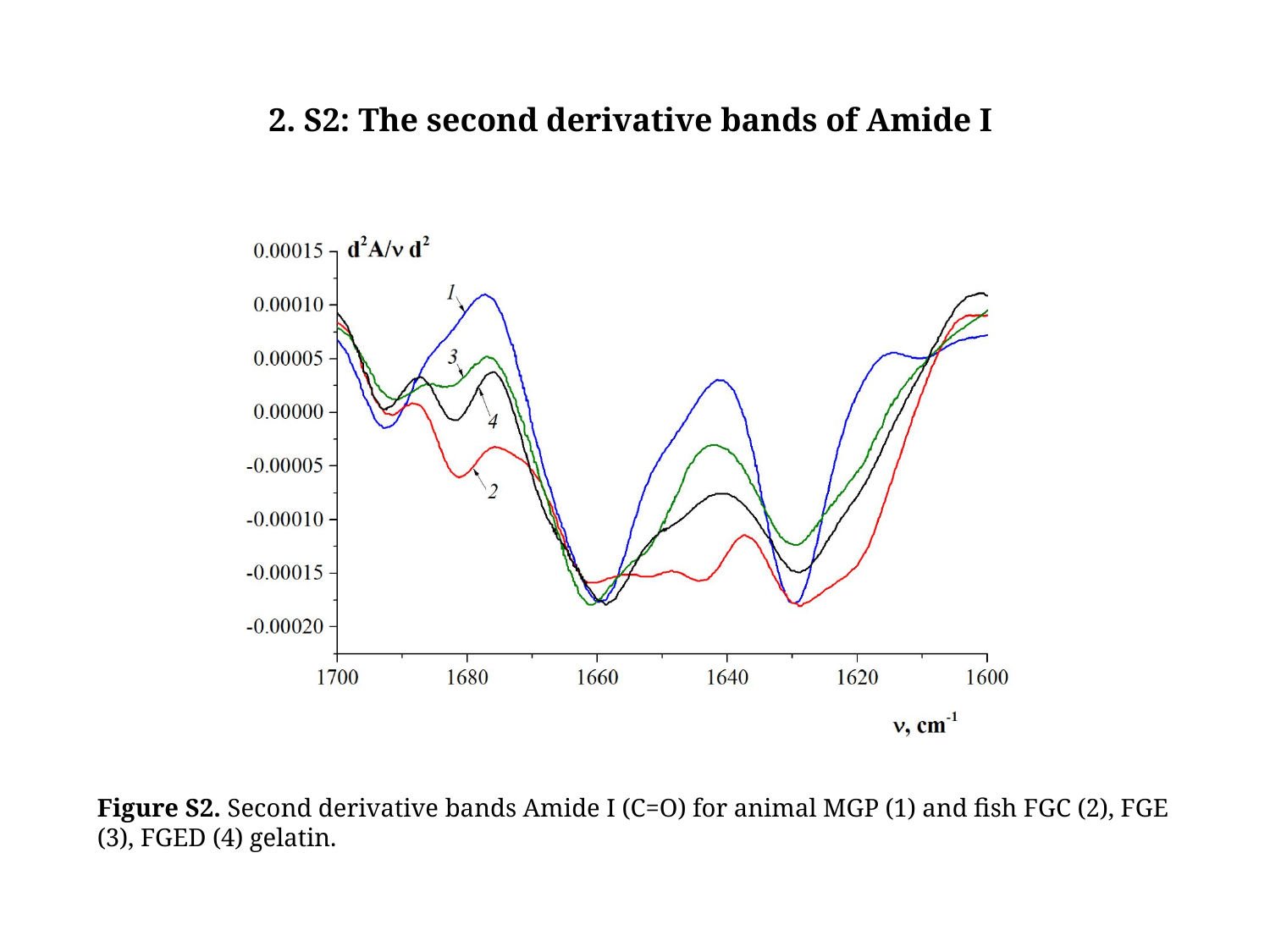

2. S2: The second derivative bands of Amide I
Figure S2. Second derivative bands Amide I (C=O) for animal MGP (1) and fish FGC (2), FGE (3), FGED (4) gelatin.

## Slide 3
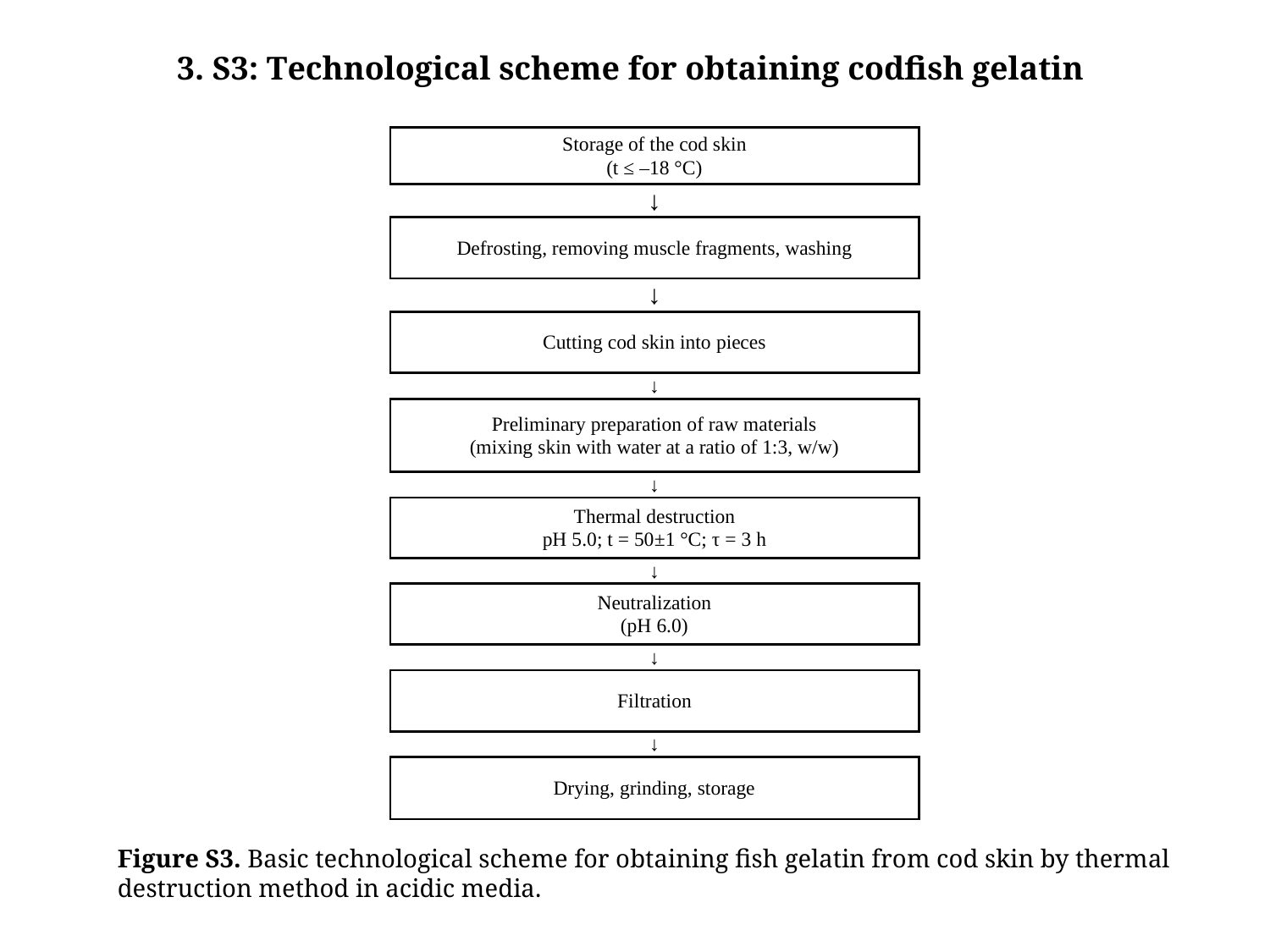

3. S3: Technological scheme for obtaining codfish gelatin
Figure S3. Basic technological scheme for obtaining fish gelatin from cod skin by thermal destruction method in acidic media.
